# Identification of sex-specific urinary biomarkers for major depressive disorder by combined application of NMR- and GC–MS-based metabonomics

**DOI:** 10.1038/tp.2016.188

**Published:** 2016-11-15

**Authors:** P Zheng, J-J Chen, C-J Zhou, L Zeng, K-W Li, L Sun, M-l Liu, D Zhu, Z-H Liang, P Xie

**Affiliations:** 1 Department of Neurology, The First Affiliated Hospital of Chongqing Medical University, Chongqing, China; 2 Institute of Neuroscience and the Collaborative Innovation Center for Brain Science, Chongqing Medical University, Chongqing, China; 3 Chongqing Key Laboratory of Neurobiology, Chongqing Medical University, Chongqing, China; 4 Institute of Life Sciences, Chongqing Medical University, Chongqing, China; 5 Department of Neurology, The Inner Mongolia Autonomous Region People's Hospital, Hohhot, Inner Mongolia, China

## Abstract

Women are more vulnerable to major depressive disorder (MDD) than men. However, molecular biomarkers of sex differences are limited. Here we combined gas chromatography–mass spectrometry (GC–MS)- and nuclear magnetic resonance (NMR)-based metabonomics to investigate sex differences of urinary metabolite markers in MDD, and further explore their potential of diagnosing MDD. Consequently, the metabolite signatures of women and men MDD subjects were significantly different from of that in their respective healthy controls (HCs). Twenty seven women and 36 men related differentially expressed metabolites were identified in MDD. Fourteen metabolites were changed in both women and men MDD subjects. Significantly, the women-specific (m-Hydroxyphenylacetate, malonate, glycolate, hypoxanthine, isobutyrate and azelaic acid) and men-specific (tyrosine, *N*-acetyl-d-glucosamine, *N*-methylnicotinamide, indoxyl sulfate, citrate and succinate) marker panels were further identified, which could differentiate men and women MDD patients from their respective HCs with higher accuracy than previously reported sex-nonspecific marker panels. Our findings demonstrate that men and women MDD patients have distinct metabonomic signatures and sex-specific biomarkers have promising values in diagnosing MDD.

## Introduction

Major depressive disorder (MDD) affects about 20% of the population during their lifetime, and accounts for ~10% of global burden of disease.^1^ The prevalence of women MDD subjects is an approximately twofold higher than that in men subjects.^2^ Previously, several hypotheses have attempted to explain this phenomenon, such as sex differences in hormones,^3^ hypothalamicpituitary-adrenal axis^4^ and immune response.^5^ However, none of these hypotheses has been universally accepted, sex-dependent molecular signatures are required to clarify.

Metabonomics enables unbiasedly measuring small-molecule metabolites in various biosamples such as urine and plasma,^6^ which has been increasingly used to identify novel disease-specific signatures as potential biomarkers.^7, 8, 9^ Nuclear magnetic resonance (NMR) spectroscopy, liquid chromatography–mass spectroscopy (LC–MS) and gas chromatography–mass spectroscopy (GC–MS) are the three major analytical techniques of metabonomics, and are suitable for non-targeted metabonomic mapping. Combined application of multiple metabolomic approaches can greatly enlarge the coverage range,^10^ and detect more comprehensive potential biomarkers than using a single approach.^11^

Previously, using NMR and GC-MS, we identified some candidate urinary diagnostic metabolite biomarkers for MDD.^12, 13^ Other researchers have also found potential biomarkers for diagnosing MDD and its subtypes.^14, 15^ However, none of these studies have considered sex differences in depression, which may to some extent limit the general applicability of these candidate biomarkers. Indeed, sex-dependent peripheral markers for MDD were increasingly emerged. For example, Owens *et al.*^16^ found that elevated morning cortisol is a stratified population-level biomarker for men MDD subjects with high depressive symptoms. Wang *et al.*^17^ reported that plasma galanin level is a potential biomarker for MDD severity, especially in women patients. These findings suggest that sex may have a vital role in molecular heterogeneity of MDD, which is required to systematically assess.

Here to identify potential sex-specific biomarkers for MDD patients, we used a combined GC–MS and NMR spectroscopic-based metabonomic approach coupled with multivariate pattern recognition techniques to profile first-episode drug-naive MDD patients (women and men, respectively) and healthy controls (HCs). In addition, drug-treated MDD patients were used to preliminarily investigate the influence of antidepressants on urinary metabolites. Our findings may contribute to future development of a sex-specific diagnostic test for MDD, and provide insight into the mechanisms associated with MDD pathogenesis in both men and women.

## Materials and methods

### Subject recruitment

For this study, HCs and MDD patients were, respectively, recruited from the Medical Examination Center and Psychiatric Center of the First Affiliated Hospital of Chongqing Medical University (Chongqing, China). This study was approved by the Ethical Committee of Chongqing Medical University. All recruited subjects provided written informed consent. The Diagnostic and Statistical Manual of Mental Disorders, 4th edition criteria (DSM-IV) for MDD was used to diagnose MDD. MDD patients with Hamilton Depression Rating Scale (HDRS) values >17 were recruited. Candidates with one of following status was excluded: (i) patients had any pre-existing physical diseases, psychiatric comorbidities or other mental disorders; (ii) patients had illicit drug use or alcohol abuse; (iii) pregnant patients. Patients with bipolar disorder were excluded by experienced psychiatrists with systematically interviewing.

The sample size chosen was mainly referred to our previous published study.^18^ The women cohort included 43 first-episode drug-naive MDD patients and 48 HCs. In MDD patients, HDRS scores ranged from 18 to 33, and the average HDRS score was 24.3. The men cohort included 50 first-episode drug-naive MDD patients and 75 HC. In MDD patients, HDRS scores ranged from 18 to 32, and the average HDRS score was 21.8. In addition, 31 drug-treated MDD (T-MDD) patients (women/men: 19/12) were included. The antidepressants taken by T-MDD patients included citalopram, duloxetine, fluoxetine, mirtazapine, paroxetine, sertraline and venlafaxine. After treatment, there were five remitters, 16 responders and 10 non-responders. The detailed clinical information was described in Table 1.

### Urinary sample collection

After overnight fasting, morning urine samples from MDD patients and HC were collected in a sterile cup, and quickly transferred to sterile tubes. All collected samples were immediately centrifuged (1500 *g* for 10 min) and the supernatants stored at −80 °C in equal aliquots until analysis.

### NMR acquisition

After thawing, urine samples were centrifuged (1500 *g* for 10 min) to remove precipitation, and then 500 μl mixed with phosphate buffer (100 μl, pH 7.4) composed of 90% heavy water (deuterium oxide, D_2_O), 3 mm sodium azide and 3 mm 3-trimethylsilyl-1-[2,2,3,3-^2^H4] propionate. Next, supernatants were obtained by centrifugation (12 000 r.p.m. for 10 min) and 500 μl transferred into 5 mm NMR tubes. NMR analysis was performed according to our previously published study.^13^ Briefly, the proton spectra were collected using a Bruker AVANCE II 600 spectrometer (Rheinstetten, Germany) operating at 600.13 MHz 1H frequency. A standard 1-dimensional (1D) pulse sequence was used. Typically, 64 transients and 16K data points were collected with a spectral width of 8000 Hz, an acquisition time of 0.945 s, and a relaxation delay of 2 s

### GC–MS acquisition

Ten microliter 0.02 mg ml^−1^ l-leucine-^13^C_6_ (internal standard solution) was added to 15 μl urine. After vortexing, 15 μl urease was added to degrade urea, and the samples incubated at 37 °C for 1 h. The mixture was then successively extracted using 240 μl and 80 μl of ice-cold methanol. After vortexing (30 s) and centrifugation (14 000 r.p.m. for 5 min at 4 °C), 224 μl of each supernatant was transferred to glass vials and vacuum-dried at room temperature. The obtained, dried metabolic extracts were derivatized using 30 μl 20 mg ml^−1^ methoxyamine at 37 °C for 1.5 h. Next, 30 μl N,O-bis-(trimethylsilyl)- trifluoroacetamide with 1% trimethylchlorosilane was added. The mixture was heated at 70 °C for 1 h to form trimethylsilyl derivatives. Finally, 1 μl derivative was injected into the GC–MS system. GC–MS analysis was performed according to our previously published study.^12^ Briefly, each 1 μl of the derivative solution was injected into an Agilent 7980 GC system (Agilent Technologies, Santa Clara, CA, USA). An HP-5 MS fused silica capillary column was used for the separation. The MS quadrupole temperature was set at 150 °C, and the ion source temperature was set at 230 °C. Data acquisition was carried out in the full scan mode from *m*/*z* 50 to 550.

### Statistical analysis

Metabolite levels were first normalized to creatinine and then unit variance scaled. The transformed data met the assumptions of the tests, and the variance between the groups was similar. Next, SIMCA-P software 11.0 was used to perform orthogonal partial least-squares discriminant analysis (OPLS-DA). A 199-iteration permutation test was applied to rule out separation non-randomness between MDD and HC samples. Metabolites with variable importance plots (VIP) >1.0 were identified as differential metabolites responsible for sample differentiation.^19^ Heat maps of these metabolites were obtained using R software 3.1.0 (Stanford University, CA, USA). In order to identify the simplest potential diagnostic biomarker panel from these differential metabolites, SPSS software 19.0 (IBM Analytics, New York, NY, USA) was used to perform binary logistic regression analysis. The Bayesian information criterion was applied to select the best-fit model.^12^ Receiver-operating characteristic analysis was applied to determine the diagnostic performance of the best-fit model.

## Results

### Different metabolite signature between women and men MDD patients

**Figure 1:**
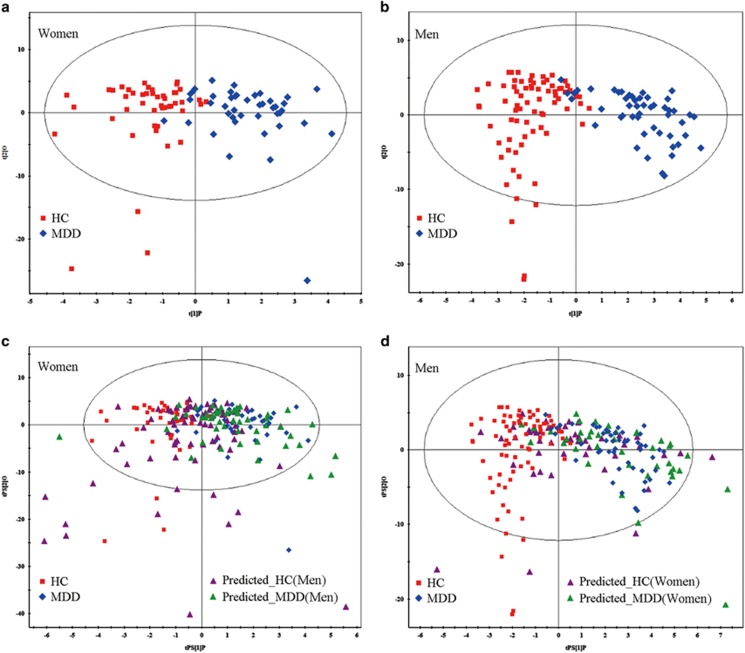
Metabonomic analysis of urine samples from women and men MDD patients vs HCs. (**a, b**) OPLS-DA score plots displaying discrimination between women and men MDD patients from their respective HCs. (**c**) The OPLS-DA model generating with women metabolite signature was used to predict class membership of men population, showing 45.3% HCs was wrongly predicted as MDD patients. (**d**) OPLS-DA model generating with men metabolite signature to predict class membership of women population, showing 56.25% HCs was wrongly designated as MDD patients. HCs, healthy controls; MDD, major depressive disorder; OPLS-DA, orthogonal partial least-squares discriminant analysis.

Metabolites from first-episode drug-naive MDD patients and HCs (women: 43 MDD vs 48 HCs; men: 50 MDD vs 75 HCs) were used to perform OPLS-DA analysis. OPLS-DA score plots showed distinct separations of both women and men MDD patients from their respective HCs with little overlap (women: R^2^Y cum=0.654, *Q*^2^=0.414; men: R^2^Y cum=0.768, *Q*^2^=0.516; Figures 1a and b). Specifically, positive R^2^Y and *Q*^2^ values indicate robust metabolic differences between MDD patients and HCs. Furthermore, the 199-iteration permutation test suggested that the two constructed OPLS-DA models were valid and positive (Supplementary Figure 1). To assess specificity of the models, the OPLS-DA model generating with women metabolite signature was used to predict class membership of men population. The T-predicted scatter plot demonstrated that 50 men MDD patients could not be effectively separated from 75 HCs, showing 45.3% HCs were wrongly predicted as MDD patients (Figure 1c). While the OPLS-DA model generating with men metabolite signature to predict class membership of women population, a similar result was obtained, showing 56.25% HCs were wrongly designated as MDD patients (Figure 1d). The findings suggest that urinary metabolite signature of depression is sex-specific at some extent.

**Figure 2:**
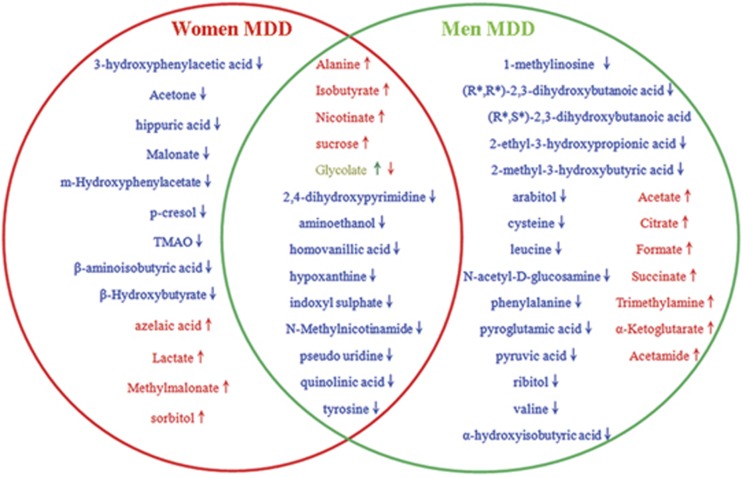
Differences and similarities of the differential metabolites in men and women MDD patients related to their corresponding healthy controls. MDD, major depressive disorder.

By analyzing the OPLS-DA loading coefficient plot, we identified 27 differential metabolites (VIP>1.0) that distinguished women MDD patients from women HCs. Compared with HCs, women MDD patients were characterized by higher levels of sorbitol, methylmalonate, lactate, isobutyrate, alanine, azelaic acid, nicotinate and sucrose, as well as lower levels of β-hydroxybutyrate, TMAO, *N*-methylnicotinamide, m-Hydroxyphenylacetate, malonate, glycolate, acetone, 2,4-dihydroxypyrimidine, 3-hydroxyphenylacetic acid, aminoethanol, hippuric acid, homovanillic acid, hypoxanthine, indoxyl sulfate, *p*-cresol, pseudo uridine, quinolinic acid, tyrosine and β-aminoisobutyric acid. A heat map of these metabolites is presented in Supplementary Figure 2. Furthermore, we identified 36 differential metabolites (VIP>1.0) that distinguished men MDD patients from men HCs. Compared with HC, men MDD patients were characterized by higher levels of 12 metabolites and lower levels of 24 metabolites. A heat map of these metabolites is presented in Supplementary Figure 3. Among the changed metabolites in both women and men MDD patients, 14 metabolites including alanine, isobutyrate, nicotinate, sucrose, glycolate, 2,4-dihydroxypyrimidine, aminoethanol, homovanillic acid, hypoxanthine, indoxyl sulfate, *N*-methylnicotinamide, pseudo uridine, quinolinic acid, tyrosine were changed in both women and men MDD (Figure 2). All these metabolite expect glycolate were consistently changed, providing clues to uncover the comment molecular basis of MDD.

### Simplified sex-specific metabolite biomarker panel

**Figure 3:**
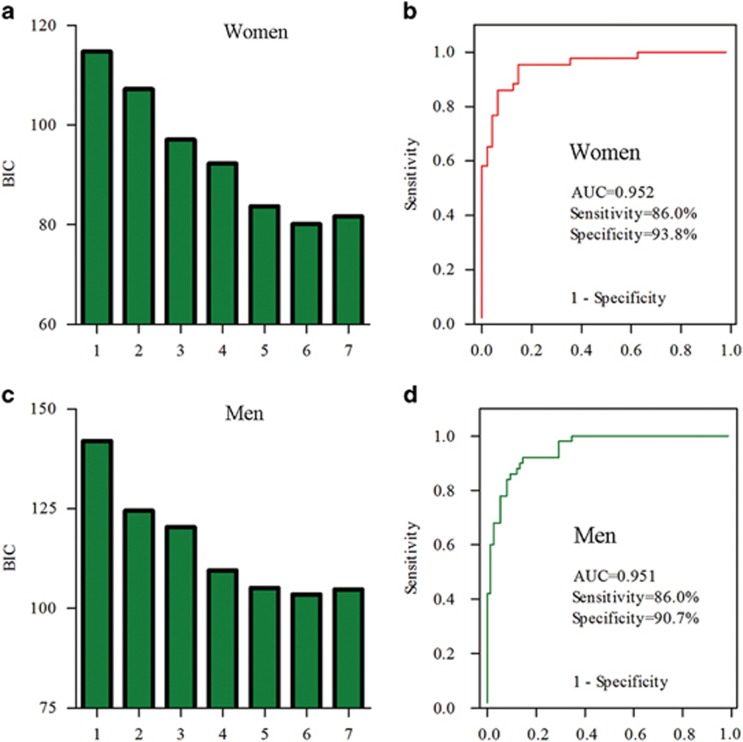
Identification and validation of the two sex-specific biomarker panels. Binary logistic regression analysis was used to simplify the candidate metabolite markers (**a–c**). Receiver-operating characteristic (ROC) analysis showing excellent diagnostic performances of these sex-specific biomarkers: the area under the curve (AUC) values of women and men marker panel was 0.952 and 0.951, respectively (**b**, **d**).

**Figure 4:**
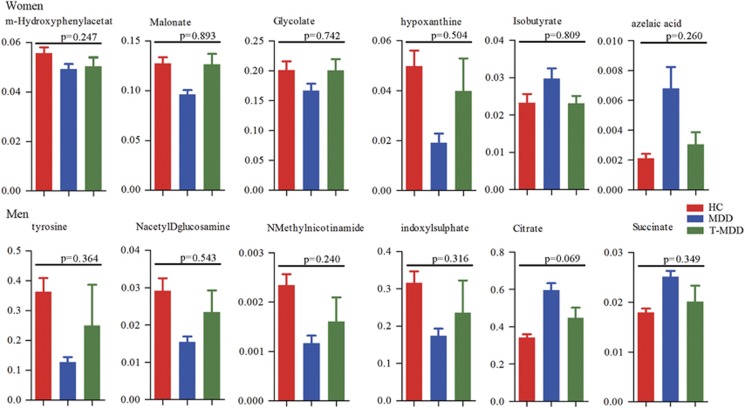
Relative concentrations of sex-specific biomarkers in HCs, MDD and treated responded or remitted MDD patients (T-MDD). There were no differences of these sex-specific biomarkers between HCs and remitted or responded MDD patients. HCs, healthy controls; MDD, major depressive disorder; T-MDD, drug-treated MDD.

Simultaneously measuring 27 or 36 urinary metabolites to diagnose MDD is not very convenient or economical in clinical practice. Therefore, binary logistic regression analysis was performed to simplify the candidate metabolite markers. We found that a women-specific biomarker panel consistent of six metabolites (m-hydroxyphenylacetate, malonate, glycolate, hypoxanthine, isobutyrate and azelaic acid) could describe the most significant deviations between women MDD patients and HCs (Figure 3a). Similarly, a men-specific biomarker panel including six metabolites (tyrosine, *N*-acetyl-d-glucosamine, *N*-methylnicotinamide, indoxyl sulfate, citrate and succinate) could describe most significant deviations between men MDD patients and HCs (Figure 3c). Furthermore, we found, after antidepressant treatments, the levels of these sex-specific markers were not significantly different between HCs and remitted or responded MDD patients. These results demonstrated that these potential sex-specific biomarkers were robust and prognostic (Figure 4).

Using these simplified sex-specific metabolite biomarker panel to construct the logistical models, we found that women panel yielded an average accuracy of 90.1% in the women population, significantly higher (*P=*0.005) than the average accuracy of 75.8% obtained using the men panel to predict the women population. Although the men panel yielded an average accuracy of 88.8% in the men population, significantly higher (*P=*0.036) than the average accuracy of 79.0% obtained using the women panel to predict the men population.

Receiver-operating characteristic analysis was further used to quantify the diagnostic performance of both panels. The area under the curve was 0.952 and 0.951 for women and men-specific metabolite biomarker panel, respectively. Sensitivity and specificity of the women panel was 86.0% and 93.8%, respectively. Although sensitivity and specificity of the men panel was 86.0% and 90.7%, respectively (Figures 3b and d). Diagnostic performance of both panels was comparable to the women and men OPLS-DA models constructed using the complete 27 and 36 differential metabolites, demonstrating the efficacy of the simplified sex-specific metabolite panel in MDD detection.

### Excellent diagnostic performance of sex-specific biomarkers

The other urinary metabolite biomarkers of MDD we previously identified by GC-MS^12^ (specifically, sorbitol, uric acid, azelaic acid, quinolinic acid, hippuric acid and tyrosine) were used to predict the women and men populations. We found that these biomarkers yielded an average accuracy of 81.3% in the women population and 76.3% in the men population. Next, the urinary metabolite biomarkers of MDD previously identified by NMR^13^ (specifically, lanine, formate, m-hydroxyphenylacetate, malonate and *N*-methylnicotinamide) were used to predict the women and men populations. We found that these biomarkers yielded an average accuracy of 76.7% in the women population and 78.6% in the men population. Overall, these results indicate that the average accuracy obtained using these biomarkers are much lower than those obtained using the sex-specific biomarkers (women: 90.1% men: 88.8%).

## Discussion

We believe this study is the first to combine NMR- and GC–MS-based metabonomics to identify potential sex-specific biomarkers for MDD. We identified 27 and 36 differential metabolites that can discriminate women and men MDD patients from HCs, respectively. Significantly, we successfully identified women- (m-hydroxyphenylacetate, malonate, glycolate, hypoxanthine, isobutyrate and azelaic acid) and men-specific (tyrosine, *N*-acetyl-d-glucosamine, *N*-methylnicotinamide, indoxyl sulfate, citrate and succinate) MDD metabolite markers, which enabled discriminating MDD patients from HCs with an area under the curve of 0.952 (women) and 0.951 (men). The diagnostic accuracy of the two sex-specific marker panels was higher than previously identified sex-nonspecific biomarkers,^12, 13^ highlighting the promising potential in diagnosis of MDD.

Currently, it is widely accepted that women are more vulnerable to MDD than men. Here, by analyzing urinary metabolite signature of MDD, we found that the woman MDD metabolic signatures were significantly different from that in man. Among the differentially expressed metabolites in women and man MDD subjects, 13 metabolites were particularly changed in women MDD patients, 22 metabolites were particularly changed in men MDD patients. Fourteen metabolites were significantly changed in both women and men MDD patients. The function of these changed metabolites were relatively diverse, which reflects the physiopathologic heterogeneity of MDD. For example, some metabolites such isobutyrate, indoxyl sulfate and tyrosine may be linked with dysbiosis of the gut microbiome, which was consistent with a new study showing depression was characterized by disturbed microbial genes and host metabolites involved in carbohydrate and amino-acid metabolism.^20^ In addition, some metabolites were linked with classic hypothesis: change of quinolinic acid may reflect disturbed kynurenine pathway in depression.^21^

To uncover the molecular basis of MDD, we used MetaboAnalyst 3.0 to examine the metabolic pathways significantly affected by these differential metabolites.^22^ Two metabolic pathways (hsa00240 and hsa00072) were significantly affected in women MDD patients (*P*<0.05; false discovery rate, FDR<0.10; Supplementary Table 1). Seven differential metabolites were involved in these two pathways with five metabolites significantly changed in women MDD patients only. Twelve metabolic pathways in men MDD patients were significantly affected (*P*<0.05; FDR<0.10; Supplementary Table 1). Sixteen differential metabolites were involved in these pathways, with nine metabolites significantly changed in men MDD patients only. Ingenuity pathway analysis was used to determine the biological function(s) influenced by these differential metabolites (Supplementary Figure 4). Although some biological functions (for example, growth of organism and proliferation of cells) had the same change (inhibited) in both men and women MDD patients, other biological functions had a different change (Supplementary Figure 5). Maes *et al.*^23^ found that serum high-density lipoprotein cholesterol and cholesterol are significantly lower in MDD patients, especially in depressed men with suicidal attempts. However, Olusi *et al.*^24^ found a significantly higher high-density lipoprotein cholesterol in MDD patients. Here we found that lipid concentration is reduced in men MDD patients, but increased in women MDD patients. Fatty acids (FAs) are the main components of neuronal membranes, determining membrane fluidity and peroxidizability.^25^ Metabolism of dietary FAs and their relationship with the hypothalamicpituitary-adrenal axis are altered in depression.^26^ Hamazaki *et al.*^27^ reported that depression may be characterized by very specific FA compositions in certain brain areas. Here we found that FA uptake may be increased in men MDD patients, although FA concentration may be increased in women MDD patients.

Of 12 sex-specific metabolite markers for MDD, one women-specific (m-hydroxyphenylacetate) and two men-specific markers (*N*-methylnicotinamide and indoxyl sulfate) are uniquely produced by gut microbita. Interestingly, some metabolites originating from gut microbiota such as hippuric acid, and trimethylamine-*N*-oxide were only significantly changed in women bipolar disorder patients.^18^ Previously, we had showed that dysbiosis of the gut microbiome may have a causal role in the development of depression.^20, 28^ Here this valuable clue is a new advance in this field, suggesting that gut microbiota may play role in the sex differences in MDD. Further studies collecting the fecal samples to analyzing the microbial composition are required to assure this hypothesis.

In addition, oxidative stress was usually linked with pathogenesis of MDD.^29^ Here we found that sorbitol and azelaic acid were significantly increased in women MDD subjects, and cysteine and 1-methylinosine were significantly decreased in men MDD subjects. There metabolites were associated with oxidative stress. In addition, some researchers found that lipid metabolism had a close relationship with MDD.^30^ Similarly, we found that the concentration of lipid was decreased in men, but increased in women. These clinical findings suggest that changes of oxidative stress status and lipid metabolism may have role in the sex differences in MDD, which are required to further clarity by basic experiments.

Monitoring levels of metabolites in biological samples are beneficial for clinical practice. For example, Miura *et al.*^31^ reported that plasma homovanillic acid may be a useful indicator to switch treatment strategies in schizophrenia. Here we found that, after antidepressant treatment, the levels of these sex-specific markers were not significantly different between HCs and remitted or responded MDD patients. This preliminary finding suggests the feasibility of using these sex-specific biomarkers to monitor the treatment response in MDD.

The limitations of our study should be acknowledged. First, as the recruited MDD subjects were divided into different groups by sex. Thus, the sample sizes were relatively small. The use of larger samples to validate the sex-specific biomarkers is required, which is an essential step prior to moving ahead to clinical practice. Second, all subjects were of the same ethnicity and were recruited from the same site. Thus, ethno- and site-specific biases cannot be ruled out. Third, only urine metabolites were analyzed. Further studies to collect cerebrospinal fluid or serum samples from MDD patients should be performed, to further ensure these urinary biomarkers physiologically relate to sex disparities in MDD. Fourth, as MDD subjects were treated with diverse antidepressants, further studies collecting urine samples from MDD subjects in randomized controlled trials are required to confirm the prognostic characteristics of these specific biomarkers.

In conclusion, by combining NMR- and GC-MS-based metabonomics, we have identified different urinary metabolic signatures between men and women MDD patients. The sex-specific urinary metabolite biomarkers for diagnosing MDD were identified, which can differentiate men and women MDD patients from their respective HCs with higher accuracy than sex-nonspecific panels identified using a single metabonomic platform. These results will be beneficial for future studies to examine differential pathogenesis between men and women MDD patients. Furthermore, they may lead to identification of sex-specific urine-based diagnostic and prognostic tests.

## Figures and Tables

**Table 1:** Clinical detail of MDD subjects and HC

	*Women*			*Men*			*T-MDD*	
	*HC*	*MDD*	*P*^a^	*HC*	*MDD*	*P*^a^	*Women*	*Men*
Sample size	48	43	—	75	50	—	19	12
Age (years)^b^	32.5±10.5	35.7±10.3	0.15	32.5±10.5	29.7±9.5	0.14	34.9±9.2	33.5±8.4
BMI^b^	21.5±2.7	20.6±2.3	0.12	21.5±2.8	22.4±2.7	0.08	21.3±2.5	24.4±3.2
HDRS scores^b^	—	24.3±4.8	—	—	21.8±4.0	—	26.3±3.3	25.2±4.9

Abbreviations: BMI, body mass index; HC, healthy controls; HDRS, Hamilton Depression Rating Scale; MDD, major depressive disorder; T-MDD, drug-treated MDD.

a Two-tailed student *t*-test.

b Values expressed as means±s.d.

## References

[bib1] Kessler RC, Berglund P, Demler O, Jin R, Koretz D, Merikangas KR et al. The epidemiology of major depressive disorder: results from the National Comorbidity Survey Replication (NCS-R). JAMA 2003; 289: 3095–3105. 12813115 10.1001/jama.289.23.3095

[bib2] Bromet E, Andrade LH, Hwang I, Sampson NA, Alonso J, de Girolamo G et al. Cross-national epidemiology of DSM-IV major depressive episode. BMC Med 2011; 9: 90. 21791035 10.1186/1741-7015-9-90 PMC3163615

[bib3] Young E, Korszun A. Sex, trauma, stress hormones and depression. Mol Psychiatry 2010; 15: 23–28. 19773810 10.1038/mp.2009.94

[bib4] Kudielka BM, Kirschbaum C. Sex differences in HPA axis responses to stress: a review. Biol Psychol 2005; 69: 113–132. 15740829 10.1016/j.biopsycho.2004.11.009

[bib5] Pitychoutis PM, Papadopoulou-Daifoti Z. Of depression and immunity: does sex matter? Int J Neuropsychopharmacol 2010; 13: 675–689. 20478108 10.1017/S1461145710000465

[bib6] Nicholson JK, Lindon JC, Holmes E. 'Metabonomics': understanding the metabolic responses of living systems to pathophysiological stimuli via multivariate statistical analysis of biological NMR spectroscopic data. Xenobiotica 1999; 29: 1181–1189. 10598751 10.1080/004982599238047

[bib7] Nicholson JK, Lindon JC. Systems biology: metabonomics. Nature 2008; 455: 1054–1056. 18948945 10.1038/4551054a

[bib8] He Y, Yu Z, Giegling I, Xie L, Hartmann AM, Prehn C et al. Schizophrenia shows a unique metabolomics signature in plasma. Transl Psychiatry 2012; 2: e149. 22892715 10.1038/tp.2012.76 PMC3432190

[bib9] Yang J, Chen T, Sun L, Zhao Z, Qi X, Zhou K et al. Potential metabolite markers of schizophrenia. Mol Psychiatry 2013; 18: 67–78. 22024767 10.1038/mp.2011.131 PMC3526727

[bib10] Bouatra S, Aziat F, Mandal R, Guo AC, Wilson MR, Knox C et al. The human urine metabolome. PloS ONE 2013; 8: e73076. 24023812 10.1371/journal.pone.0073076 PMC3762851

[bib11] Chen JJ, Liu Z, Fan SH, Yang DY, Zheng P, Shao WH et al. Combined application of NMR- and GC-MS-based metabonomics yields a superior urinary biomarker panel for bipolar disorder. Sci Rep 2014; 4: 5855. 25068480 10.1038/srep05855 PMC5376169

[bib12] Zheng P, Chen JJ, Huang T, Wang MJ, Wang Y, Dong MX et al. A novel urinary metabolite signature for diagnosing major depressive disorder. J Proteome Res 2013; 12: 5904–5911. 24224655 10.1021/pr400939q

[bib13] Zheng P, Wang Y, Chen L, Yang D, Meng H, Zhou D et al. Identification and validation of urinary metabolite biomarkers for major depressive disorder. Mol Cell Proteomics 2013; 12: 207–214. 23111923 10.1074/mcp.M112.021816 PMC3536901

[bib14] Nichkova MI, Huisman H, Wynveen PM, Marc DT, Olson KL, Kellermann GH. Evaluation of a novel ELISA for serotonin: urinary serotonin as a potential biomarker for depression. Anal Bioanal Chem 2012; 402: 1593–1600. 22160204 10.1007/s00216-011-5583-1

[bib15] Ding X, Yang S, Li W, Liu Y, Li Z, Zhang Y et al. The potential biomarker panels for identification of Major Depressive Disorder (MDD) patients with and without early life stress (ELS) by metabonomic analysis. PloS ONE 2014; 9: e97479. 24870353 10.1371/journal.pone.0097479 PMC4037179

[bib16] Owens M, Herbert J, Jones PB, Sahakian BJ, Wilkinson PO, Dunn VJ et al. Elevated morning cortisol is a stratified population-level biomarker for major depression in boys only with high depressive symptoms. Proc Natl Acad Sci USA 2014; 111: 3638–3643. 24550453 10.1073/pnas.1318786111 PMC3948242

[bib17] Wang YJ, Yang YT, Li H, Liu PZ, Wang CY, Xu ZQ. Plasma galanin is a biomarker for severity of major depressive disorder. Inte J Psychiatry Med 2014; 48: 109–119. 10.2190/PM.48.2.d 25377152

[bib18] Chen JJ, Huang H, Zhao LB, Zhou DZ, Yang YT, Zheng P et al. Sex-specific urinary biomarkers for diagnosing bipolar disorder. PloS ONE 2014; 9: e115221. 25531985 10.1371/journal.pone.0115221 PMC4274077

[bib19] Qi Y, Li P, Zhang Y, Cui L, Guo Z, Xie G et al. Urinary metabolite markers of precocious puberty. Mol Cell Proteomics 2012; 11: M111 011072. 10.1074/mcp.M111.011072 PMC3270102 22027199

[bib20] Zheng P, Zeng B, Zhou C, Liu M, Fang Z, Xu X et al. Gut microbiome remodeling induces depressive-like behaviors through a pathway mediated by the host's metabolism. Mol Psychiatry 2016; 21: 786–796. 27067014 10.1038/mp.2016.44

[bib21] Reus GZ, Jansen K, Titus S, Carvalho AF, Gabbay V, Quevedo J. Kynurenine pathway dysfunction in the pathophysiology and treatment of depression: Evidences from animal and human studies. J Psychiatr Res 2015; 68: 316–328. 26028548 10.1016/j.jpsychires.2015.05.007 PMC4955923

[bib22] Xia J, Sinelnikov IV, Han B, Wishart DS. MetaboAnalyst 3.0—making metabolomics more meaningful. Nucleic Acids Res 2015; 43: W251–W257. 25897128 10.1093/nar/gkv380 PMC4489235

[bib23] Maes M, Smith R, Christophe A, Vandoolaeghe E, Van Gastel A, Neels H et al. Lower serum high-density lipoprotein cholesterol (HDL-C) in major depression and in depressed men with serious suicidal attempts: relationship with immune-inflammatory markers. Acta Psychiatr Scand 1997; 95: 212–221. 9111854 10.1111/j.1600-0447.1997.tb09622.x

[bib24] Olusi SO, Fido AA. Serum lipid concentrations in patients with major depressive disorder. Biol Psychiatry 1996; 40: 1128–1131. 8931915 10.1016/S0006-3223(95)00599-4

[bib25] Bazinet RP, Laye S. Polyunsaturated fatty acids and their metabolites in brain function and disease. Nat Rev Neurosci 2014; 15: 771–785. 25387473 10.1038/nrn3820

[bib26] Mocking RJ, Verburg HF, Westerink AM, Assies J, Vaz FM, Koeter MW et al. Fatty acid metabolism and its longitudinal relationship with the hypothalamic-pituitary-adrenal axis in major depression: Associations with prospective antidepressant response. Psychoneuroendocrinology 2015; 59: 1–13. 26010860 10.1016/j.psyneuen.2015.04.027

[bib27] Assies J, Mocking RJ, Lok A, Ruhe HG, Pouwer F, Schene AH. Effects of oxidative stress on fatty acid- and one-carbon-metabolism in psychiatric and cardiovascular disease comorbidity. Acta Psychiatr Scand 2014; 130: 163–180. 24649967 10.1111/acps.12265 PMC4171779

[bib28] Wong ML, Inserra A, Lewis MD, Mastronardi CA, Leong L, Choo J et al. Inflammasome signaling affects anxiety- and depressive-like behavior and gut microbiome composition. Mol Psychiatry 2016; 21: 797–805. 27090302 10.1038/mp.2016.46 PMC4879188

[bib29] Michel TM, Pulschen D, Thome J. The role of oxidative stress in depressive disorders. Current Pharm Des 2012; 18: 5890–5899. 10.2174/138161212803523554 22681168

[bib30] Liu X, Zheng P, Zhao X, Zhang Y, Hu C, Li J et al. Discovery and validation of plasma biomarkers for major depressive disorder classification based on liquid chromatography-mass spectrometry. J Proteome Res 2015; 14: 2322–2330. 25784130 10.1021/acs.jproteome.5b00144

[bib31] Miura I, Shiga T, Katsumi A, Kanno-Nozaki K, Mashiko H, Niwa S et al. Switching antipsychotics to aripiprazole or blonanserin and plasma monoamine metabolites levels in patients with schizophrenia. Hum Psychopharmacol 2014; 29: 199–202. 24590545 10.1002/hup.2386

