# Supplementary material for: Identification of sex-specific urinary biomarkers for major depressive disorder by combined application of NMR- and GC–MS-based metabonomics
Source: Transl Psychiatry. 2016 Nov 15;6(11):e955–. doi: 10.1038/tp.2016.188 (PMC5314113; doi:10.1038/tp.2016.188)

**Identification of sex-specific urinary biomarkers for diagnosing major depressive disorder by combined application of NMR- and GC-MS-based metabonomics**

Peng Zheng, Jian-jun Chen, Chan-juan Zhou, Li Zeng,, Ke-wei Li, , Lin Sun,,

Mei-ling Liu, Dan Zhu, Zi-hong Liang, Peng Xie

Supplementary Table 1 Significantly affected metabolic pathways

| **KEGG pathway** | **Total** | **Hits** | **P-value** | **FDR** | **Compound name** |
| --- | --- | --- | --- | --- | --- |
| *Women* |  |  |  |  |  |
| hsa00240 | 60 | 5 | 0.0002 | 0.0192 | Malonate, 2,4-dihydroxypyrimidine,pseudo uridine, Methylmalonate, β-aminoisobutyric acid |
| hsa00072 | 6 | 2 | 0.0013 | 0.0558 | Acetone, β-Hydroxybutyrate |
| *Men* |  |  |  |  |  |
| hsa00430 | 20 | 4 | 0.0000 | 0.0036 | Cysteine, Alanine, Pyruvic acid, Acetate |
| hsa00970 | 75 | 6 | 0.0001 | 0.0036 | phenylalanine, Cysteine, valine, Alanine, leucine, Tyrosine |
| hsa00630 | 50 | 5 | 0.0001 | 0.0036 | Citrate, Succinate, Pyruvic acid, Glycolate, Formate |
| hsa00770 | 27 | 4 | 0.0001 | 0.0036 | 2,4-dihydroxypyrimidine, Cysteine, valine, Pyruvic acid |
| [hsa00020](http://www.genome.jp/dbget-bin/www_bget?pathway:hsa00020) | 20 | 3 | 0.0012 | 0.0182 | Citrate, Succinate, Pyruvic acid |
| hsa00360 | 45 | 4 | 0.0013 | 0.0182 | phenylalanine, tyrosine, Succinate, Pyruvic acid |
| hsa00250 | 24 | 3 | 0.0021 | 0.0248 | Alanine, Pyruvic acid, Succinate |
| hsa00290 | 27 | 3 | 0.0030 | 0.0307 | Pyruvic acid, valine, leucine |
| hsa00620 | 32 | 3 | 0.0050 | 0.0447 | Pyruvic acid, Formate, Acetate |
| hsa00910 | 39 | 3 | 0.0087 | 0.0674 | Formate, phenylalanine, Tyrosine |
| hsa00350 | 76 | 4 | 0.0092 | 0.0674 | tyrosine, Pyruvic acid, Succinate, homovanillic acid |
| hsa00760 | 44 | 3 | 0.0122 | 0.0818 | quinolinic acid, Nicotinate, pyruvic acid |

Abbreviations: Hits, the number of significantly changed metabolites in this pathway; FDR, false discover rate; hsa00240, Pyrimidine metabolism; hsa00072, Synthesis and degradation of ketone bodies; hsa00430, Taurine and hypotaurine metabolism; hsa00970, Aminoacyl-tRNA biosynthesis; hsa00630, Glyoxylate and dicarboxylate metabolism; hsa00770, Pantothenate and CoA biosynthesis; [hsa00020](http://www.genome.jp/dbget-bin/www_bget?pathway:hsa00020), Citrate cycle (TCA cycle); hsa00360, Phenylalanine metabolism; hsa00250, Alanine, aspartate and glutamate metabolism; hsa00290, Valine, leucine and isoleucine biosynthesis; hsa00620, Pyruvate metabolism; hsa00910, Nitrogen metabolism; hsa00350, Tyrosine metabolism; hsa00760, Nicotinate and nicotinamide metabolism.

**Supplementary Figure 1** The 199- iteration permutation test for men and women OPLS-DA model.

**
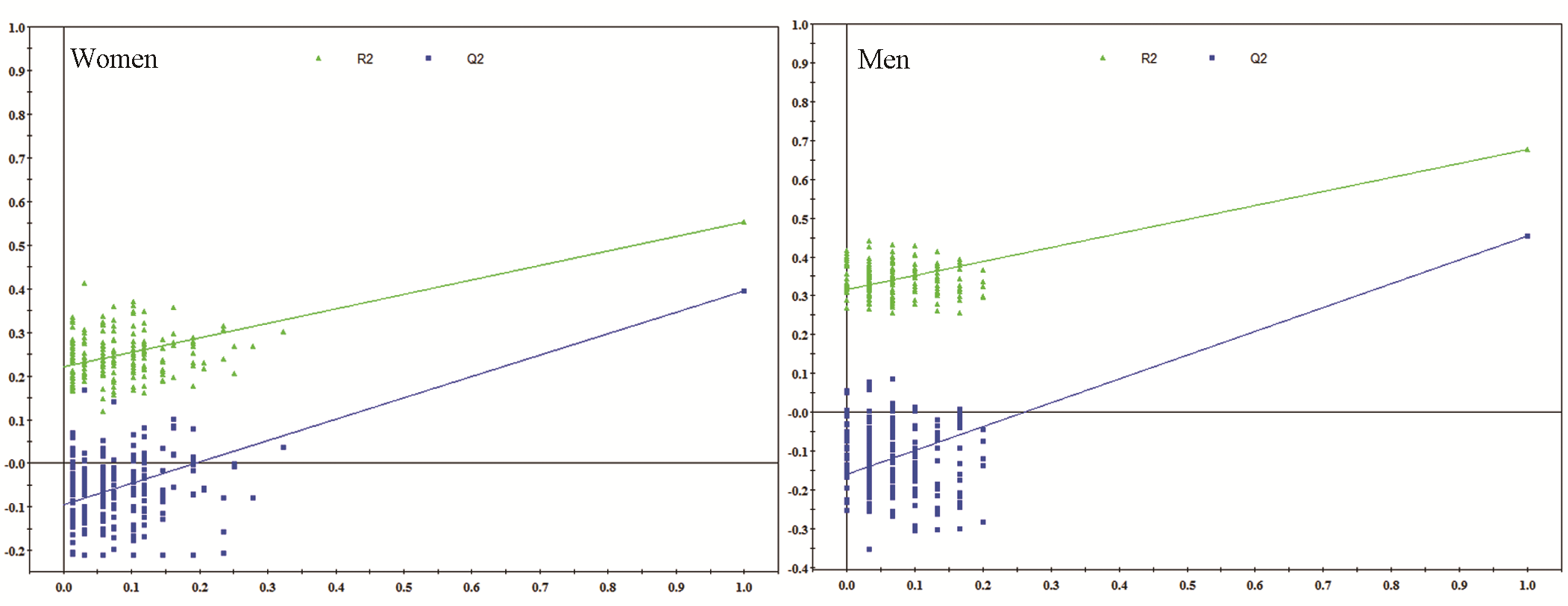
**

**Supplementary Figure 2** Heatmap of the differential metabolites in women MDD patients relate to women healthy controls.


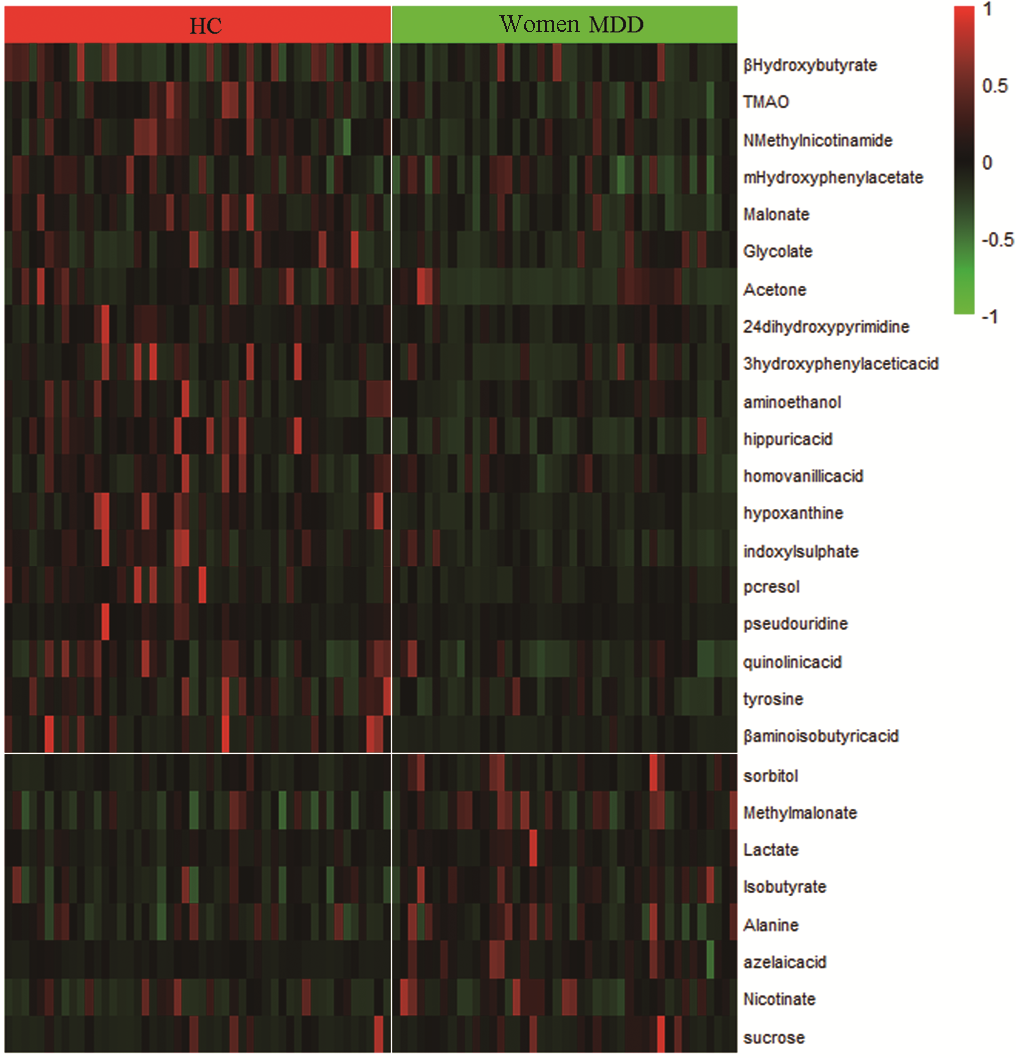


**Supplementary Figure 3** Heatmap of the differential metabolites in men MDD patients relate to men healthy controls.


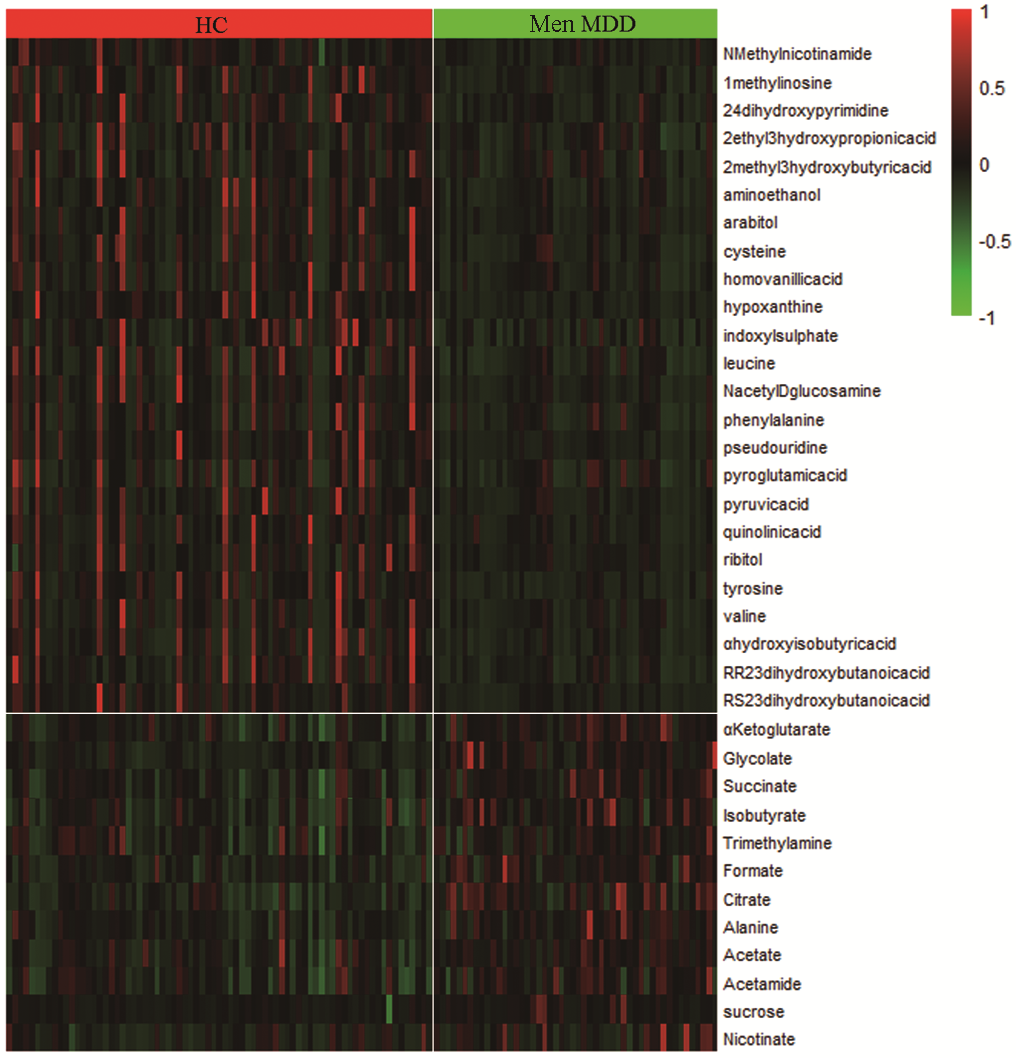


**Supplementary Figure 4** Biological function inhibited and activated in men and women MDD patients. Inhibited and activated are depicted by red and green colors, respectively.


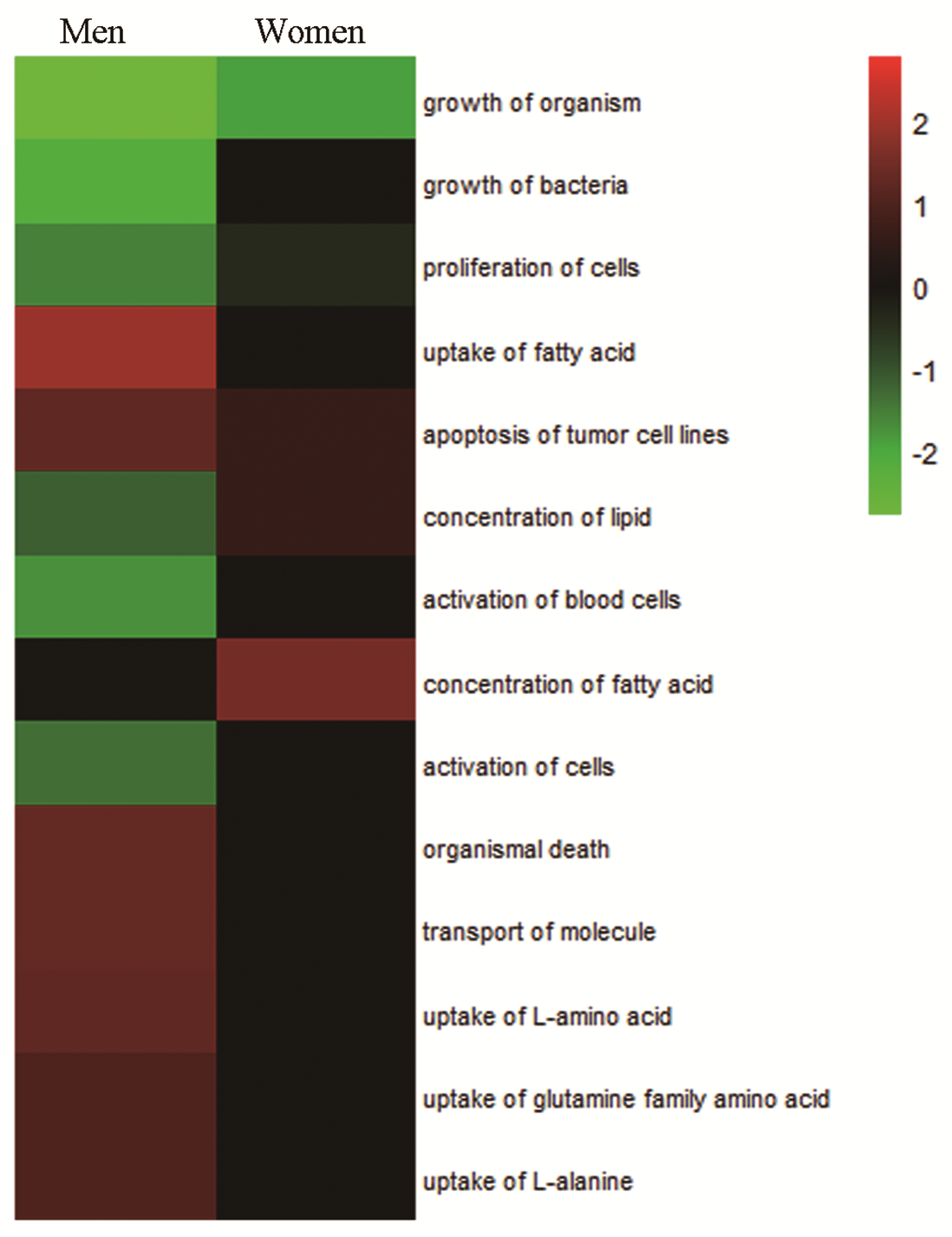


**Supplementary Figure 5** Several biological functions. Blue and orange dot line with arrow indicated activation. Blue and orange dot line with vertical line indicated inhibition. Gray dot line with arrow indicated not predicted interaction. Yellow dot line with arrow or vertical line indicated the findings inconsistent with state of downstream molecule.


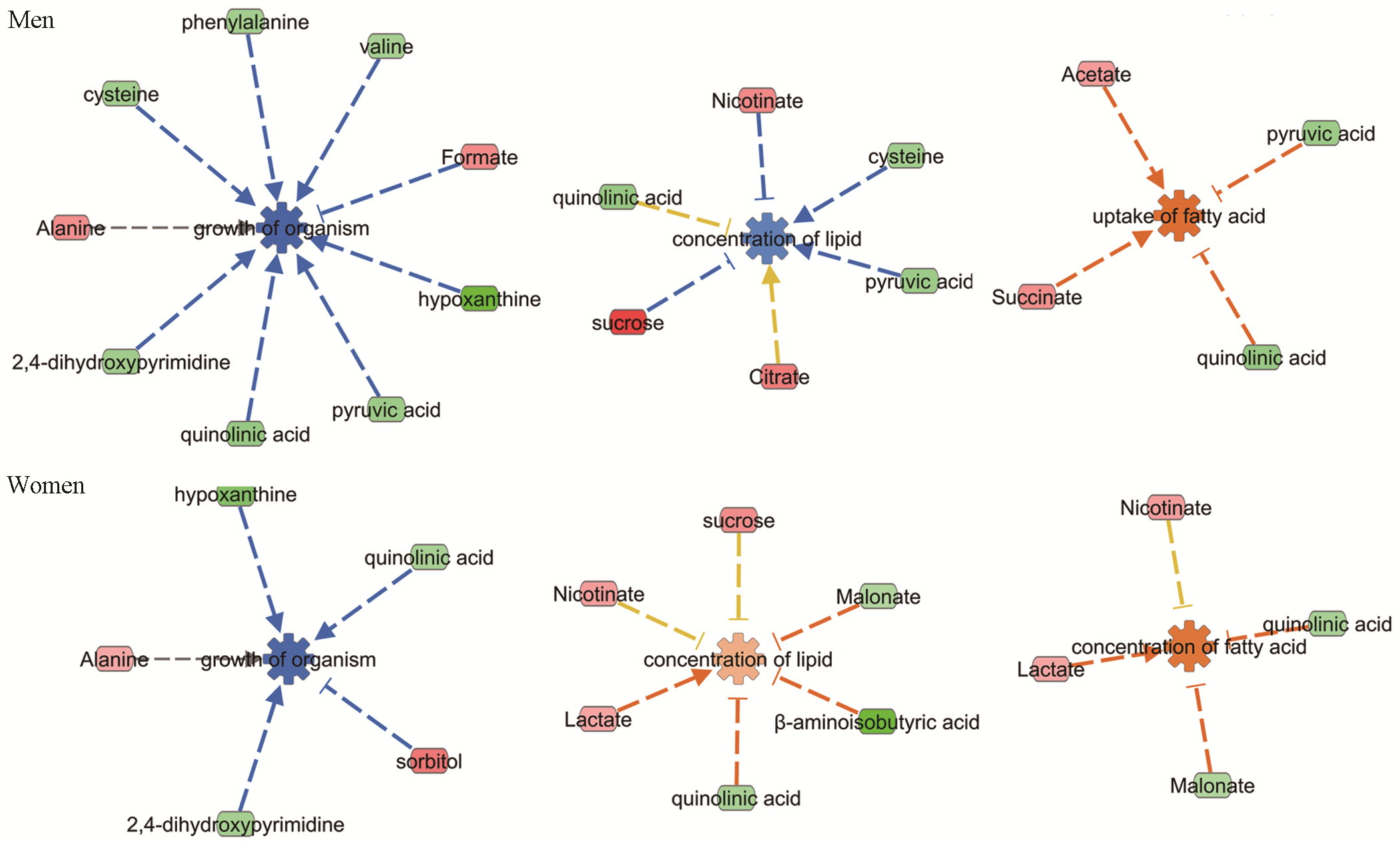

Supplement: Supplementary Materials [file tp2016188x1.doc]
